# Expression of miRNAs miR-133b and miR-206 in the *Il17a/f* Locus Is Co-Regulated with IL-17 Production in αβ and γδ T Cells

**DOI:** 10.1371/journal.pone.0020171

**Published:** 2011-05-26

**Authors:** Jan D. Haas, Kiran Nistala, Franziska Petermann, Namita Saran, Vijaykumar Chennupati, Susanne Schmitz, Thomas Korn, Lucy R. Wedderburn, Reinhold Förster, Andreas Krueger, Immo Prinz

**Affiliations:** 1 Institute for Immunology, Hannover Medical School, Hannover, Germany; 2 Rheumatology Unit, Institute of Child Health, University College London, London, United Kingdom; 3 Klinikum rechts der Isar, Department of Neurology, Technical University Munich, Munich, Germany; Universität Würzburg, Germany

## Abstract

Differentiation of T helper 17 cells (Th17) is a multistep process that involves the cytokines IL-6, TGF-β, and IL-23 as well as IL-1β, IL-21, and TNF-α. Thereby, robust induction of the capacity to produce IL-17 involves epigenetic modifications of the syntenic *Il17a/f* locus. Using inbred mouse strains, we identified co-regulation of gene transcription at the *Il17a/f* locus with the nearby microRNAs miR-133b and miR-206 that are clustered approximately 45 kb upstream of *Il17a/f*. Expression of these microRNAs was specific for Th17 as compared to other CD4^+^ T cell subsets and this was equally valid for *in vitro* polarized and *ex vivo* derived cells. From all factors analyzed, IL-23 was the most important cytokine for the *in vitro* induction of miR-133b and miR-206 in naive CD4^+^ T cells of wild type mice. However, analysis of IL-23R deficient mice revealed that IL-23R signaling was not essential for the induction of miR-133b and miR-206. Importantly, we found a similar co-regulation in CCR6^+^ and other γδ T cell subsets that are predisposed to production of IL-17. Taken together, we discovered a novel feature of T cell differentiation towards an IL-17-producing phenotype that is shared between αβ and γδ T cells. Notably, the specific co-regulation of miR-133b and miR-206 with the *Il17a/f* locus also extended to human Th17 cells. This qualifies expression of miR-133b and miR-206 in T cells as novel biomarkers for Th17-type immune reactions.

## Introduction

microRNAs (miRNAs) are 21–24 nucleotide long non-coding RNAs which play a critical role in the regulation of gene expression. They usually target the 3′ untranslated region (3′-UTR) of their respective target gene(s) at the mRNA level –resulting in mRNA degradation, mRNA destabilization or inhibition of translation [1].

Gene regulation by miRNAs has recently emerged to be critical for both development and proper function of the immune system. Thus, various miRNAs such as miR-155, miR-223, miR-146, miR-150, miR-181a or the miR-17∼92 cluster have been implicated in hematopoietic lineage decisions or in controlling different developmental checkpoints [2], [3]. Furthermore, miRNAs contribute to the terminal differentiation of mature lymphocytes [4], [5], [6].

CD4^+^ T helper (Th) cells orchestrate the adaptive immune response so that appropriate effector mechanisms are elicited dependent on the nature of the invading pathogen. Th1 cells are induced to produce mainly IFN-γ in order to fight viruses and intracellular bacteria, whereas Th2 cells produce IL-4/-5/-13 in response to infections by helminths and other parasites. More recently, Th17 cells have been described to secrete IL-17A, IL-17F and IL-22 to combat extracellular bacteria and fungi by stimulating epithelial cells to produce chemokines and cytokines, which drive the immune response e.g. by neutrophil influx [7], [8]. Finally, CD4^+^ T cell differentiation can also lead to the development of tolerogenic induced regulatory T cells (iTreg). Dysregulation of Th cell differentiation may result in ineffective clearance of pathogens and/or in the development of autoimmune or allergic diseases. Therefore, Th cell differentiation needs to be tightly regulated. In this context, miRNAs have been shown to be involved in the development or function of all four main T helper subsets [9], [10], i.e. miR-155 in Th1, miR-126 in Th2, miR-155 in Treg and miR-326 in Th17. For instance, deletion of a single miRNA, miR-155, can influence the fitness of Treg cells [11]. Furthermore, Ets-1, a negative regulator of Th17 differentiation, has been recently reported as a target of miR-326, the amount of which seems to be associated with disease relapse in multiple sclerosis patients [12]. However, these authors did not directly correlate the production of IL-17A, the key cytokine of Th17 cells, with disease onset and severity.

In addition to CD4^+^ Th17 cells, γδ T cells are major or even main producers of IL-17A depending on the physiological or pathological context [13]. Recently, we and others identified a subset of γδ T cells expressing the CC chemokine receptor (CCR) 6 as potent IL-17A producers upon cytokine stimulation, encounter with pathogen products or environmental cues [14], [15].

Genome-wide gene expression analysis has recently revealed that eukaryotic gene regulation is not only dependent on the direct action of transcription factors on promoter regions, but is also subject to higher order regulation dependent on the location of genes within the genome [16]. Thus, co-expression of genes depends in part on their relative distance with neighboring genes being frequently co-expressed [17], either because of shared regulatory elements or because of extended chromatin opening. Likewise, the gene for two miRNAs miR-133b and miR-206, which are probably expressed from a bicistronic pri-miRNA [18], is located directly upstream of the *Il17a* and *Il17f* (*Il17a/*f) gene locus. So far, miR-206 and miR-133b have been reported to be specifically important for muscle regeneration and development [18], [19], [20] and their expression has been suggested to be largely restricted to skeletal muscle and osteoblasts. However, based on their close proximity to the *Il17a/f* gene locus we hypothesized that expression of these two miRNAs and secretion of the main Th17 cytokine IL-17A might correlate. Here, we demonstrate elevated expression of miR-206/133b in *in vitro* polarized Th17 cells as well as in freshly isolated murine and human Th17 cells and in IL-17A producing innate lymphocytes such as CCR6-expressing γδ T cells. Furthermore, we show that amongst multiple Th17 polarizing cytokines, IL-23 was the most important one for miR-133b and miR-206 expression. Taken together, our data reveal a previously unrecognized expression of miR-206/133b in lymphocytes, which is tightly coordinated with the expression of IL-17A.

## Results

### miRNAs miR-133b and miR-206 are syntenic to the *Il17a/f* locus and are specifically expressed in Th17 cells polarized *in vitro*

**Figure 1:**
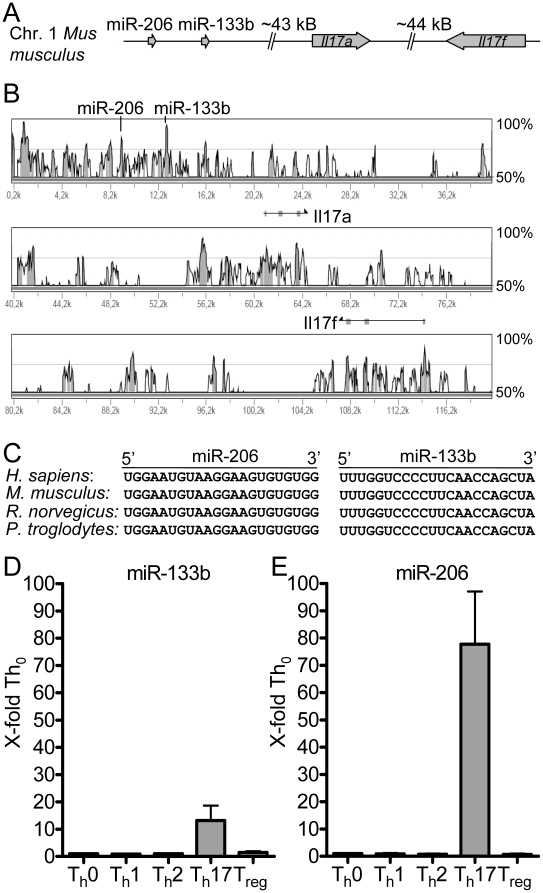
miRNAs miR-133b and miR-206 are syntenic to the IL-17A/IL-17F locus and are specifically expressed in Th17 cells polarized *in vitro*. (A) Schematic representation of the *Il17a/f* locus. *Il17a* and *Il17f* are syntenically linked in approx. 44 kB distance. The two miRNAs miR-133b and miR-206 are located in close proximity upstream to the *Il17a/f* locus. (B) VISTA plot of the mouse *Il17a/f* locus in which mouse sequence is shown on the x axis and percent similarity to human on the y axis. (C) Sequence comparison of mature miR-133b and miR-206 in H.s., M.m. R.n. and P.t. (D), (E) Spleen and peripheral lymph node cells were isolated from DO11.10 mice, co-cultured with sex-matched *BALB/c* irradiated feeder cells and polarized for the different T helper cell subset conditions (T_h_0, T_h_1, T_h_2, T_h_17 and T_reg_). Expression levels of miR-133b (D) and miR-206 (E) were analyzed by qRT-PCR. Values are plotted as fold difference compared to non-polarized cells (T_h_0). Error bars show ±SEM of n≥3 experiments with 2–3 mice per experiment.

Many miRNAs have been found to be clustered and are likely to be co-expressed when less than 50 kb apart [21]. miR-133b and miR-206 form such a cluster with a distance of approximately 4 kb between the coding sequences of the two mature miRNAs. We found that in the murine genome the miR-206/133b cluster lies in close proximity (less than 50 kb upstream) to the genes coding for the cytokines IL-17A and IL-17F (Fig. 1A), which themselves are organized syntenically in a head-to-head direction and probably arose through gene duplication.

The miR-206/133b cluster is present in all vertebrate genomes analyzed so far [22]. Performing whole genome alignment using VISTA [23] we tested whether the proximity of miR-206/133b to the *Il17a/f* locus is similarly conserved. This analysis revealed that indeed miR-206/133b is syntenic to the *Il17a/f* locus in mouse and humans (Fig. 1B) as well as in rat and chimpanzee (data not shown). Furthermore, the mature miRNA sequences of miR-133b and miR-206 are identical between mouse, human, rat and chimpanzee (Fig. 1C).

Genome-wide expression analysis has revealed that neighboring genes are frequently co-expressed [17] suggesting that the miR-206/133b cluster and IL-17A/F might be co-expressed as well. To test this hypothesis, we isolated CD4^+^ T cells from transgenic mice expressing the DO11.10 T cell receptor specific for ovalbumin (amino acids 323–339). Cells were stimulated under neutral conditions (Th0) or polarized *in vitro* towards the Th1, Th2, Th17 and Treg lineages. Expression of miR-133b and miR-206 was assessed using qRT-PCR. Polarization towards Th17 resulted in a 13-fold increase of miR-133b expression when compared to Th0 cells, whereas polarization into other CD4^+^ T cell lineages did not alter miR-133b expression (Fig. 1D). Similarly, elevated miR-206 expression (78-fold relative to Th0) was only detected in Th17, but not in Th1, Th2 or Treg polarized cells (Fig. 1E). However, additional stimulation of Th17-polarized cells with PMA/ionomycin did not influence the expression of miR-133b and miR-206 (Fig. S1). Together, these results indicate that the two miRNAs of the miR-206/133b cluster are indeed co-regulated together and with the neighboring IL-17 locus.

### Th17 cells express elevated amounts of miR-133b and miR-206 *in vivo*

**Figure 2:**
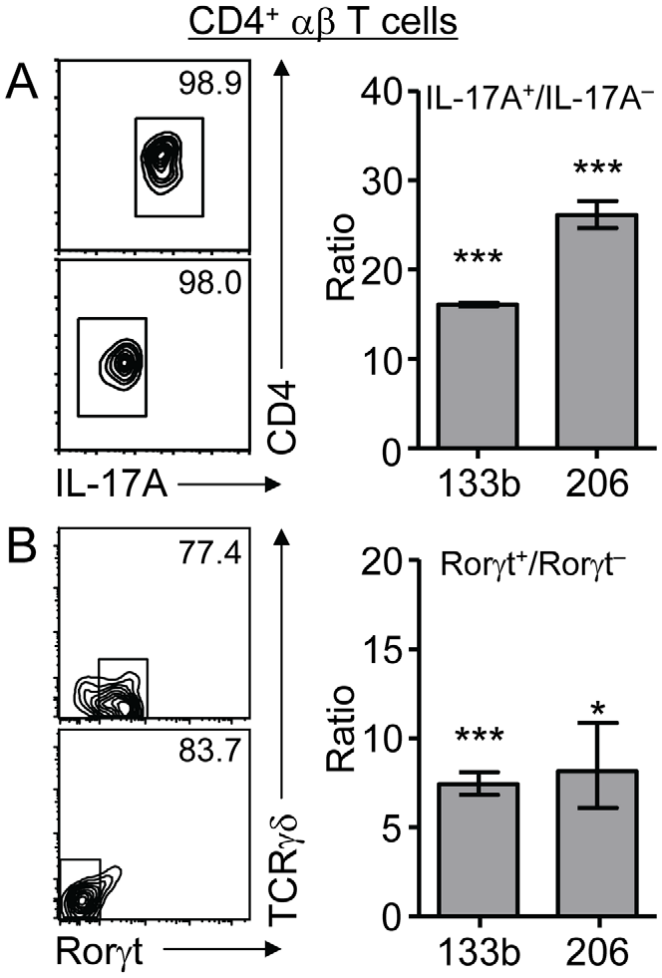
IL-17 producing *in vivo* polarized CD4^+^ Th17 cells express elevated amounts of miR-133b and miR-206. (A) CD4^+^ T cells from *C57BL/6* mice were sorted into IL-17A^+^ and IL-17A^−^ populations and analyzed for expression of miR-133b and miR-206 by qRT-PCR. Dot plots show post-sort analysis of one representative experiment. (B) CD4^+^ T cells were sorted into GFP^+^ (Rorγt^+^) and GFP^−^ (Rorγt^−^) populations from heterozygous Rorγt reporter mice and analyzed as in (A). Dot plots show post-sort analysis of one representative experiment. Values are plotted as fold increase ( = ratio) compared to the respective negative population. The graphs show representative experiments from n = 3 independent experiments with similar results. Error bars represent SD values of triplicates from one experiment with 4–6 mice.

Although *in vitro* polarization recapitulates the phenotypes of various Th lineages, *in vitro* and *in vivo* polarized cells may not necessarily be identical. Therefore, we assessed miR-133b and miR-206 expression levels in freshly isolated T cells. To this end, CD4^+^ T cells were sorted *ex vivo* based on IL-17A expression as assessed using an IL-17A secretion assay (Fig. 2A, right panels). Consistent with data obtained from *in vitro* polarized cells, IL-17A secreting cells expressed 16-fold higher levels of miR-133b and 26-fold higher levels of miR-206 when compared to IL-17A negative CD4^+^ T cells (Fig. 2A, left panel). Since miR-133b and, especially, miR-206 are important for muscle cell proliferation and differentiation [24], we sought to exclude the possibility that cell activation and proliferation correlated with heightened expression of these miRNAs. However, when comparing their expression in sorted activated/memory CD4^+^ T cells versus naive CD4^+^ T cells we found only a marginal upregulation of miR-206/133b in the former (<2 fold), which may reflect the few Th17 cells contained in the activated/memory CD4^+^ T cell population (Fig. S2). Furthermore, RORγt has been described as signature transcription factor for Th17 cells. Therefore, we extended our analyses of miR-206/133b expression to CD4^+^ T cells positive or negative for RORγt expression as assessed by expression of a GFP reporter gene knocked into the *Rorc* locus [25]. Low levels of GFP fluorescence intensity precluded the isolation of pure GFP-positive and GFP-negative populations (Fig. 2B, right panels). Nevertheless, both miR-133b and miR-206 expression was elevated 7-fold and 8-fold, respectively, in cells enriched for RORγt (GFP) expression when compared to RORγt (GFP)-negative cells (Fig. 2B left panel). Thus, in CD4^+^ T cells miR-133b and miR-206 are co-expressed with IL-17A *in vivo*.

### Co-expression of IL-17A and miR-206/133b in γδ T cells

**Figure 3:**
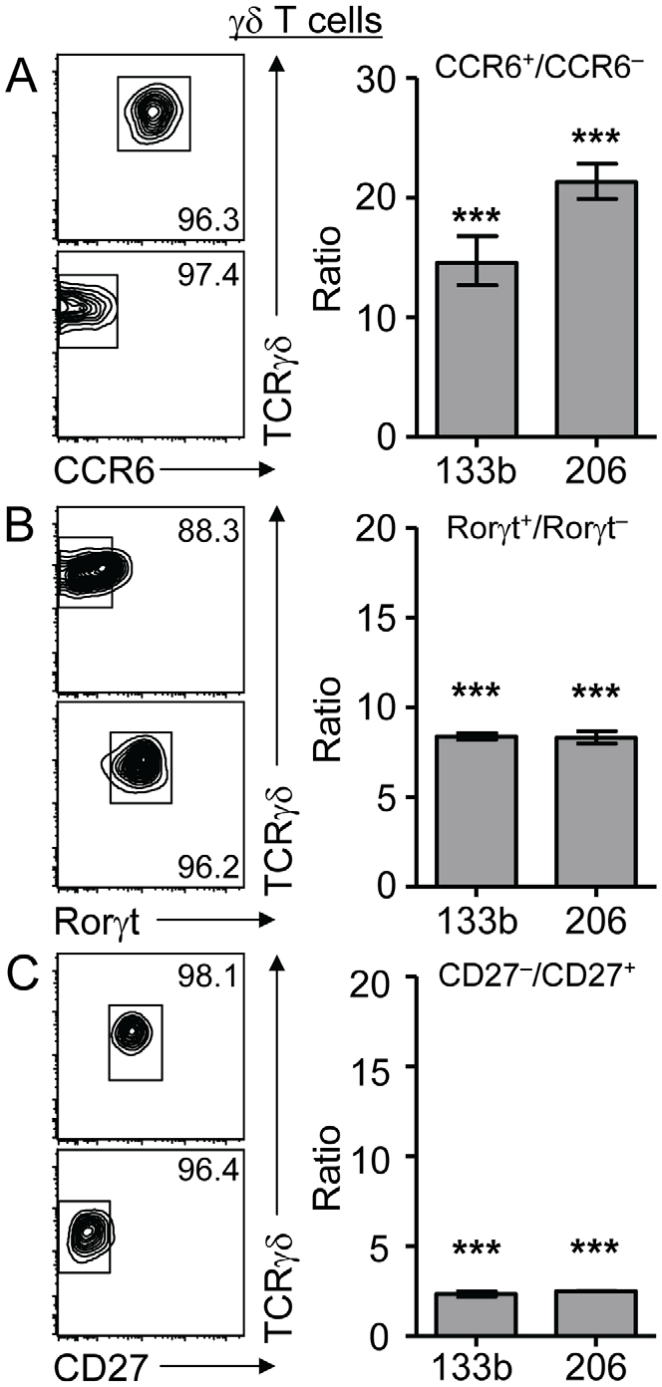
Correlation of IL-17A production and miR-206/133b expression in γδ T cells. (A) γδ T cells from *Tcrd-H2BeGFP* mice were sorted into a CCR6^+^ and CCR6^−^ populations and analyzed for expression of miR-133b and miR-206 by qRT-PCR. Dot plots show post-sort analysis of one representative experiment. (B) γδ T cells were sorted into GFP^+^ (Rorγt^+^) and GFP^−^ (Rorγt^−^) populations from heterozygous Rorγt reporter mice and analyzed as in (A). (C) γδ T cells from *Tcrd-H2BeGFP* mice were sorted into CD27^−^ and CD27^+^ populations and analyzed as in (A). Dot plots show post-sort analysis of one representative experiment. Values are plotted as fold increase ( = ratio) compared to the respective negative population. The graphs show representative experiments from n = 3 independent experiments with similar results. Error bars represent SD values of triplicates from one experiment with 4–6 mice.

Recently, evidence has emerged that innate lymphocytes such as γδ T cells constitute a major source of IL-17A [13], [26]. We and others have shown that IL-17A production in γδ T cells is largely restricted to a CC chemokine receptor (CCR) 6 expressing subpopulation [14], [15]. It was thus instrumental to test whether co-expression of miR-133b and miR-206 is restricted to CD4^+^ Th17 cells or whether it is a more general phenomenon. To this end, we isolated γδ T cells based on reporter gene expression from *C57BL/6-Tcrd-H2BeGFP* mice [27], in which all γδ T cells express high levels of GFP, and separated these cells into CCR6-positive and CCR6-negative subsets (Fig. 3A, right panels). qRT-PCR revealed that CCR6-positive γδ T cells expressed 15-fold and 21-fold higher levels of miR-133b and miR-206, respectively, when compared to CCR6-negative γδ T cells (Fig. 3A, left panel). Expression of RORγt is not only indicative of CD4^+^ Th17 cells, but also characterizes γδ T cells expressing IL-17A [26]. Therefore, we isolated GFP-positive and GFP-negative γδ T cells from RORγt reporter mice as described for Figure 2B (Fig. 3B, right panels) and assessed the expression of miR-133b and miR-206. Expression levels of both miRNAs were increased in GFP-positive cells when compared to GFP-negative cells (both approximately 8-fold). Another marker used to distinguish subsets of γδ T cells is CD27, and CD27 expression inversely correlates with the expression of IL-17A [28]. Thus, CD27 expression might serve as an additional tool to assess the correlation between miR-206/133b expression and IL-17A expression. CD27-positive and CD27-negative γδ T cells were sorted from *Tcrd-H2BeGFP* reporter mice (Fig. 3C, right panels) and expression levels of miR-133b and miR-206 were assessed by qRT-PCR. In accordance with a less stringent correlation of CD27 expression and IL-17A expression, expression levels of miR-133b and miR-206 were only slightly elevated (2-fold and 2.5-fold, respectively) in CD27-negative versus CD27-positive γδ T cells (Fig. 3C, left panel). Taken together, these data indicate that co-expression of miR-206/133b and IL-17A is not restricted to CD4^+^ Th17 cells, but also holds true for IL-17A expressing innate lymphocytes, suggesting that this co-regulation is a general phenomenon shared between αβ and γδ T cells.

### IL-23 promotes expression of miR-206/133b

**Figure 4:**
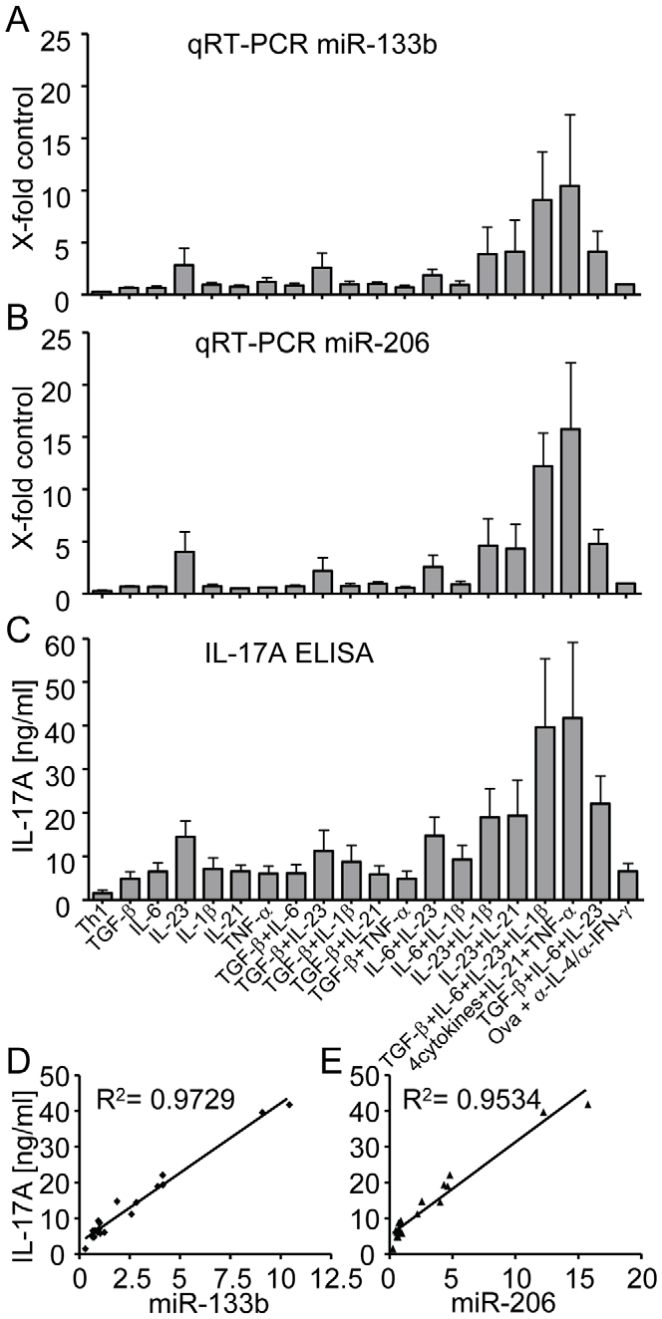
The Th17 polarizing cytokine IL-23 promotes expression of miR-206/133b as well as secretion of IL-17A. (A, B) Spleen and peripheral lymph node cells were isolated from DO11.10 mice, cocultered with sex-matched *BALB/c* irradiated feeder cells and polarized to either Th1 or treated with TGF-β, IL-6, IL-23, IL-1β, IL-21 and TNF-α in various combinations (4cytokines = TGF-β, IL-6, IL-23 and IL-1β). Values show fold increase ( = ratio) compared to cells cultured only with Ova_323–329_/antibodies. Expression levels of miR-133b (A) and miR-206 (B) were analyzed by qRT-PCR. (C) Secreted IL-17A was determined in culture supernatants of each condition from the cells in (A) and (B) by ELISA. Values show absolute amounts of IL-17A in the cell culture supernatant in ng/ml. Error bars show ±SEM of n = 3 experiments with 2–3 mice per experiment. (D), (E) Scatter plot of IL-17A protein concentration versus relative expression of miR-133b (D) and miR-206 (E) and correlation coefficient for ELISA compared to qRT-PCR.

**Figure 5:**
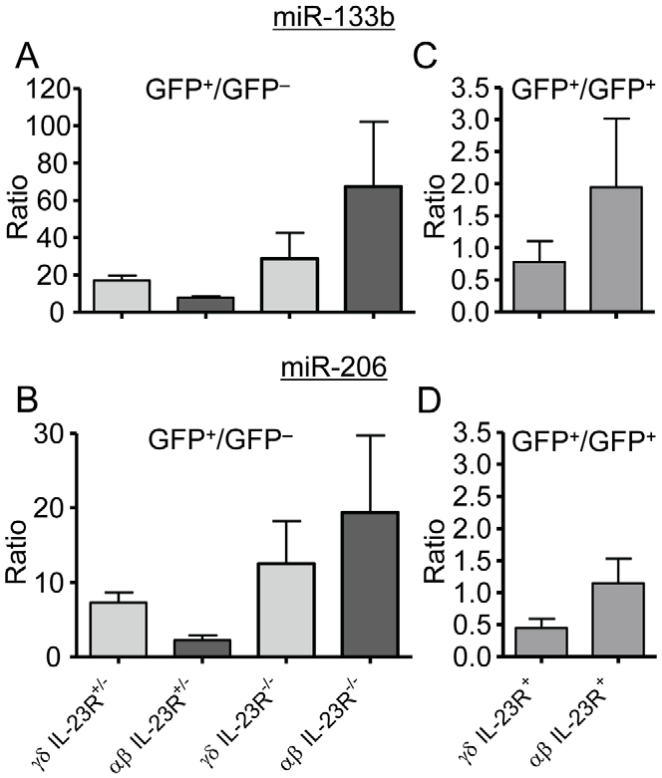
IL-23R signalling is not essential for the induction of miR-133b and mir-206. IL-23R^+^ (GFP^+^) or IL-23R^−^ (GFP^−^) γδ and αβ T cells from either homozygous (IL-23R^−/−^) or heterozygous IL-23Rgfp.KI mice were compared for their miR-133b and miR-206 expression in (A) and (B). GFP^+^ γδ and αβ T cells from heterozygous or GFP^+^ γδ and αβ T cells from homozygous IL-23Rgfp.KI mice were compared for their miR-133b and miR-206 expression in (C) and (D). Error bars show ±SEM of n = 3 experiments with cells pooled from 10 mice per group.

Th17 cell polarization can be induced *in vitro* using a combination of various cytokines such as IL-23, TGF-β, IL-6 and IL-1β. Whereas IL-6 and low amounts of TGF-β are necessary to differentiate naive CD4^+^ T cells into Th17 cells, IL-23 appears to be essential for sustained differentiation of Th17 cells [29]. However, the extent of IL-17 induction by individual cytokines may vary and thus comparing induction of IL-17 with induction of miR-206/133b by individual Th17 polarizing cytokines might provide some mechanistic insight into miR-206/133b/IL-17 co-regulation. Therefore, DO11.10 TCR transgenic CD4^+^ T cells were polarized towards Th17 using individual polarizing cytokines as well as cytokine combinations. Notably, under these conditions, addition of IL-23 alone was able to induce low levels of miR-133b and miR-206 (Fig. 4A, B) as well as IL-17A as assessed by ELISA (Fig. 4C). Furthermore, combination of two cytokines induced marked up-regulation of the two miRNAs and IL-17A secretion only when IL-23 was part of the combination and no additive effect was observed, when two other cytokines were applied. Thus, of all cytokines tested IL-23 appears to be the most important for expression of miR-133b and miR-206 under standard conditions of *in vitro* polarization. Since in these experiments IL-4 and IFN-γ but not TGF-β or other factors were blocked it is likely that traces of IL-23R inducing cytokines were derived from fetal bovine serum in the medium. This is consistent with the observation that combination of all four cytokines generally used for Th17 polarization, namely IL-23, TGF-β, IL-6 and IL-1β or the additional use of IL-21 and TNF-α resulted in a profound synergistic effect with respect to miR-133b and miR-206 upregulation when compared to IL-23 alone or a combination of two cytokines (Fig. 4A, B). IL-17A levels of secretion appeared to follow the same pattern (Fig. 4C). In order to more quantitatively assess the correlation between induction of miR-133b and miR-206 versus secretion of IL-17A dependent on the different cytokine cocktails used for polarization, we determined the respective correlation coefficients. Both miR-133b induction (Fig. 4D) as well as miR-206 induction (Fig. 4E) correlated strongly with secretion of IL-17A with correlation coefficients R^2^ of 0.97 and 0.95, respectively. Taken together, these results point to an important role of IL-23 signaling in miR-206/133b induction in lymphocytes and strengthen the hypothesis of a strict co-regulation of miR-206/133b and IL-17A. However, truly naive CD4^+^ T cells do not express IL-23R. In order to clarify the role of IL-23 for the induction of miR-206/133b *in vivo* we sorted αβ or γδ T cells from either heterozygous or homozygous IL-23R-GFP knock-out/reporter-knock-in mice [30]. We found that IL-23R expression was not required for miR-206/133b expression in IL-23R-reporter GFP^+^ cells (Fig. 5). We thus propose that IL-23 chiefly promotes the expansion and maintenance of IL-17 producing cells.

### Primary human Th17 cells, but not Th1 cells, express miR-133b and miR-206

**Figure 6:**
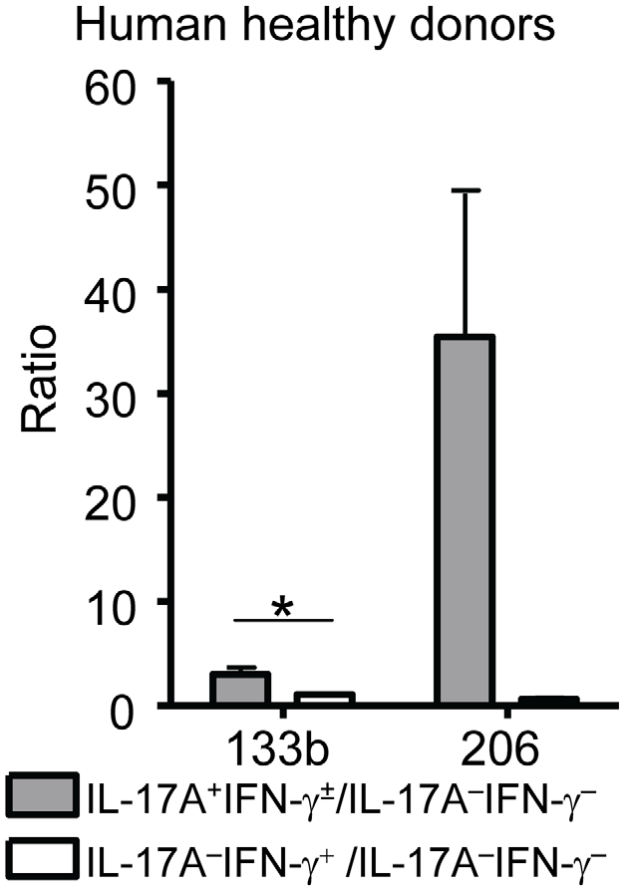
Primary human Th17 cells, but not Th1 cells, express miR-133b and miR-206. CD4^+^ T cells were isolated from peripheral blood of 3 human healthy donors, enriched using magnetic beads and then FACS sorted into 3 different populations: IL-17A^+^, IL-17A^−^ IFNγ^+^ and IL-17A^−^IFN-γ^−^. Expression levels of miR-133b and miR-206 were analyzed by qRT-PCR. Values are plotted as fold increase compared to the respective IL-17A^−^ IFN-γ^−^ population. P-value for miR-206 in IL-17A^+^ compared to IFN-γ^+^ expressing cells was 0.0645. Error bars show ±SEM for the 3 different donors.

Human Th17 cells have been suggested to be involved in diseases such as multiple sclerosis [31] or juvenile idiopathic arthritis [32], but have been described to differ in certain aspects from their murine counterparts, e.g. human Th17 cells co-express IFN-γ more often than murine Th17 cells [33]. Given these apparent differences, we tested whether miR-206/133b and IL-17 were co-regulated in human Th17 cells as well. Therefore, we isolated human primary Th17, Th1 and Th0 cells from healthy donors using a combination of IL-17 and IFN-γ cytokine secretion assays as described [34]. Although a certain degree of donor variability was observed, expression levels of miR-133b and miR-206 were consistently higher in IL-17A secreting human T cells (2 to 4-fold and 15 to 60-fold, respectively) when compared to Th0 cells (Fig. 6). In contrast, miR-206/133b levels were largely identical between human Th1 and Th0 cells. Thus, our data indicate that the miR-206/133b cluster is co-regulated with IL-17 in human T cells as well. In conclusion, we provide evidence that the two miRNAs of the miR-206/133b cluster neighboring the *Il17a/f* locus are co-regulated in murine and human lymphocytes.

### Little impact of ectopic miR-206/133b expression on Th17 differentiation

**Figure 7:**
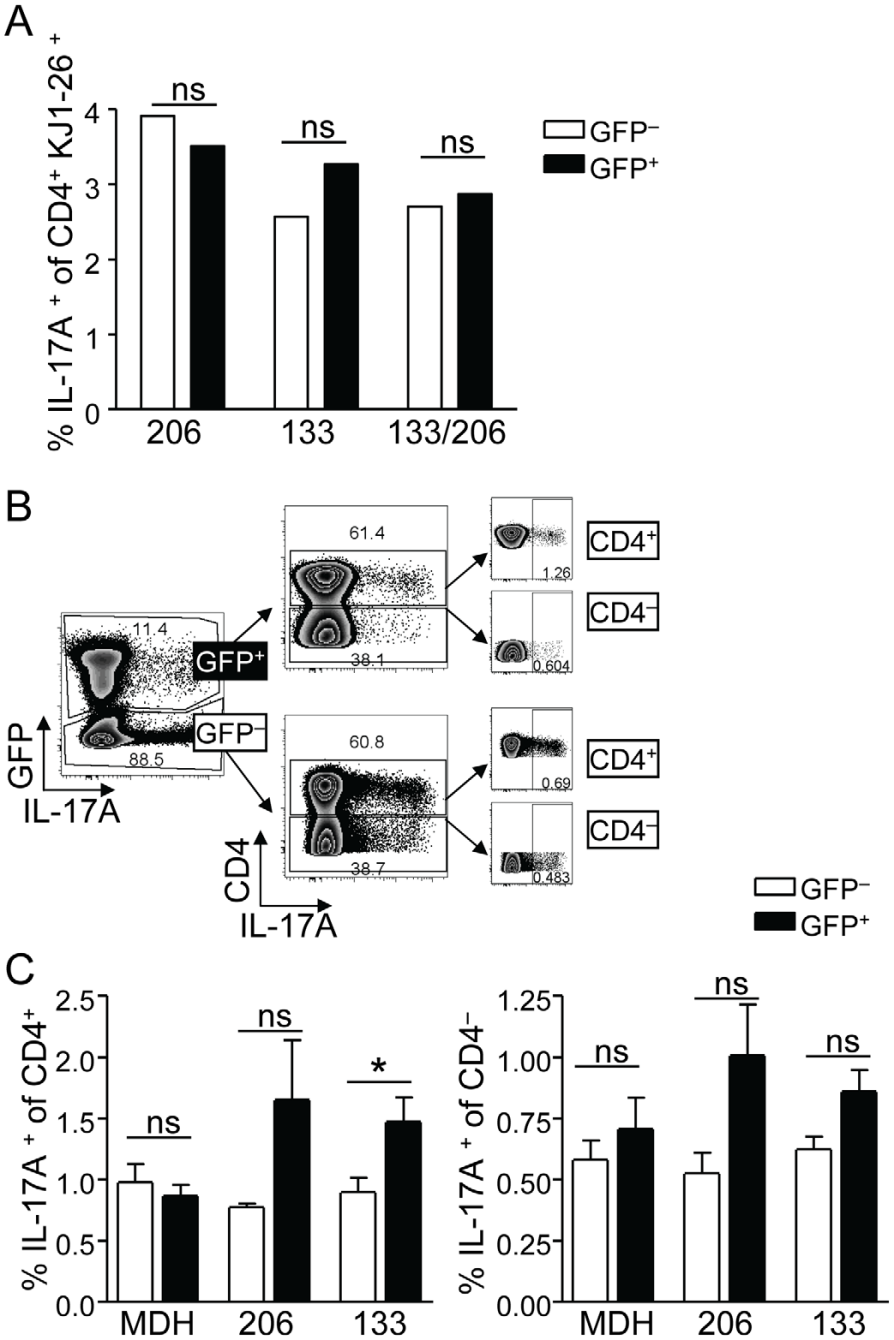
Functional outcome of ectopically expressed miR-133b and miR-206 *in vitro* and *in vivo*. (A) *In vitro* assay. MACS-enriched CD4^+^ T cells from TCR-transgenic DO.11.10 mice were retrovirally transduced with miR-133b or miR-206 or with both and stimulated under Th17 polarization inducing conditions. Frequency of IL-17 producing CD4^+^ T cells among transduced (GFP^+^) and non-transduced (GFP^−^) cells of the same well was compared. One representative of two independent experiments with similar results is shown. (B, C) Frequency of IL-17 producing cells overexpressing miR-133b or miR-206 *in vivo*. Lineage negative bone marrow was transduced with miR-133b or miR-206 or with the empty vector MDH1-PGK-GFP2.0 and served to reconstitute lethally irradiated *C57BL/6* wild type mice. After 8–10 weeks, chimeras were analyzed for the occurrence and frequency of IL-17 producing cells within the transduced GFP^+^ and non-transduced GFP^−^ lymphocytes from peripheral lymph nodes and spleen. (B) Representative gating strategy after excluding autofluorescent and B220^+^ cells. (C) Frequency of IL-17 producing CD4^+^ and CD4^−^ cells, respectively. At least 5 chimeric mice were individually analyzed for each condition.

Finally, to examine a potential functional role of the miR-206/133b cluster in Th17 polarization, we undertook a series of efforts to manipulate the expression of the two investigated miRNAs in T cells both *in vivo* and *in vitro*. To this end, we employed retroviral vectors for miRNAs miR-133b and miR-206 based on MDH1-PGK-GFP_2.0 [35]. The respective constructs were used for stable transduction of ovalbumin-specific T cells from DO11.10 mice that were previously stimulated with their cognate OVA-peptide for 24 h under non-polarizing conditions. After transduction, T cells were cultured under Th17 or other conditions for an additional 7 days. After restimulation with PMA/ionomycin, cells were fixed and stained for intracellular IL-17. IL-17 producing cells were equally frequent among transduced GFP^+^ and non-transduced GFP^−^ DO11.10 cells (Fig. 7A). Furthermore, to test a potential role of miR-133b and miR-206 in the development of IL-17 producing cells *in vivo*, we retrovirally transduced bone marrow derived hematopoietic precursors and generated bone marrow chimeras that contained transduced GFP^+^ and non-transduced GFP^−^ T cells. Although statistically not significant, we observed a trend pointing to a higher frequency of IL-17 producing cells among the CD4^+^ Th and CD4^−^ cells in chimeras from bone marrow transduced with either miR-133b or miR-206 but not the empty vector (Fig. 7B, C).

## Discussion

In this work, we revealed a tight co-regulation of the expression of the miR-206/133b cluster with the potential to produce the pro-inflammatory cytokine IL-17A in lymphocytes. This finding was equally valid for cells that had acquired the capacity to produce IL-17 cytokines *in vivo* and for *in vitro* polarized CD4^+^ Th17 cells. Furthermore, this co-regulation was similarly observed in those subsets of γδ T cells that were reported to be genuine IL-17A producers, i.e. characterized by the expression of CCR6 [14], [15] and the IL-23R [36]. However, at present it is not clear whether expression of miR-206/133b plays a functional role in Th17 lineage differentiation by targeting specific mRNAs or whether its expression may just be involved in epigenetic regulation of the *Il17a/f* locus. In fact, experiments, in which miR-206 or miR-133b or both were over-expressed in T cells showed no significant changes in the capacity of naive αβ T cells to become polarized towards Th17 or other Th lineages as well as iTreg. Similarly, over-expression of miR-206 or miR-133b in bone marrow chimeras did not reveal striking changes in the frequency of IL-17 producing CD4^+^ Th or CD4^−^ cells.

Synteny of the miR-206/133b and *Il17a/f* loci is conserved among mammals and chicken, suggesting that the transcriptional co-regulation is physiologically relevant. Along this line, genome-wide gene expression studies suggest that neighboring co-expressed genes in eukaryotes are often functionally connected, implying some operational similarity to prokaryotic operons [16]. However, despite the high expression levels of miR-206/133b we found no detectable expression of *Il17* in muscle tissue (Fig. S3) suggesting that the co-regulation of the miR-206/133b cluster and the *Il17a/f* locus is specific for T lymphocytes.

Recently, Akimzhanov et al. characterized the size of the locus control region of the *Il17a/f* genes, which extends beyond the highly conserved non-coding sequences upstream of the *Il17a/f* locus, where miR-206/133b is located [37]. Furthermore, miR-206/133b are close to a conserved noncoding sequence (CNS-1 or -60 region), which was recently reported to undergo chromatin remodeling in Th17 cell differentiation and therefore possibly provides accessibility to regulatory elements [38]. In addition to classical roles of miRNAs, namely gene-regulation by targeting 3′-UTRs of mRNA, it is thus conceivable that expression of the miRNAs miR-133b and miR-206 is involved in the regulation of *Il17a* and *Il17f* locus accessibility comparable to the involvement of miRNA in DNA methylation in plants [39]. Transcriptional activity of the miR-206/133b cluster may promote opening of the whole region including the *Il17a/f* locus. Likewise, the miR-181-a1/181-b1 cluster is located upstream of the CD45 gene locus and is known to be involved in the T versus B lineage decision in CD45^+^ lymphocytes [35].

Using quantitative Real-Time PCR (qRT-PCR), we found considerable differences of miR-206/133b expression levels between IL-17 producing and non-producing cells suggesting high specificity of the assay. Indeed, a very recent comprehensive resource study based on deep-sequencing reproduced our findings that miR-206/133b are specifically upregulated in Th17 cells compared to other T cell subsets [40]. However, overall expression levels of miR-133b in lymphocytes were much lower when compared to samples of skin and muscle tissue. Notably, miR-206 was expressed to considerable extents in Th17 cells with more than 1000 sequence tags per million. Furthermore, expression of this miRNA was only 14-fold and 19-fold lower in Th17 cells when compared to skin and muscle [40]. Accordingly, miR-206/133b were initially described to be specifically expressed in skeletal muscle as well as osteoblasts suggesting that the *Il17a/f* locus might be accessible for gene transcription in these tissues as well. So far, expression of IL-17A and/or IL-17F has not been reported for these cell types. Whereas IL-17A expression is thought to be restricted to lymphocytes, IL-17F expression has also been demonstrated in colonic epithelial cells [41]. Several targets of miR-133b and miR-206 such as DNA pol α, connexin43, histone deacetylase 4 (HDAC4) and the transcription factor Pitx3 have been reported to play a role in muscle development, regeneration of neuro-muscular synapses as well as osteoblast function [22], [42], [43], [44]. Therefore, it will be interesting to see whether such targets are also functionally regulated in IL-17A producing lymphocytes. In addition, we performed *in silico* analysis of potential target genes for both miRNAs with the target prediction tools miRANDA, TargetScan and PicTar in order to identify novel target genes of potential immunological interest. Interestingly, the gene coding for the transcription factor Ets1 was predicted to be a target of both miR-133b (one predicted recognition site) and miR-206 (four predicted recognition sites). Ets1 was previously suggested to regulate Th17 cell differentiation as a direct target of miR-326 [12]. However, we could not confirm an effect of miR-133b or miR-206 overexpression on constructs containing the 3′-UTR of the Ets1 gene in the context of the BW5147α-β- thymoma cell line (Fig. S4).

Co-regulation of neighboring genes may be controlled by multiple non-mutually exclusive factors. Thus, changes in chromatin status may occur over extended distances. Location of miR-206/133b within the reported locus control region of the *Il17a/f* gene is consistent with this hypothesis. Alternatively, neighboring genes may directly share promoter or enhancer elements. In this study we found that both miR-206/133b and IL-17A can be induced by IL-23 signaling. IL-23/p19 induced activation of STAT3 leads to direct binding of phosphorylated STAT3 to IL-17A and IL-17F promoters suggesting that, in addition to chromatin remodeling, sharing of regulatory elements may as well contribute to co-regulation of miR-206/133b and IL-17A [45]. Taken together, we discovered co-regulation of miR-133b and miR-206 with the *Il17a/f* locus as a novel feature of T cell differentiation that is shared between mouse αβ and γδ T cells and also extended to human Th17 cells. This qualifies transcription of miR-133b and miR-206 as a novel marker for T cells of an IL-17-producing phenotype.

## Materials and Methods

### Mice

Six- to ten-wk-old *C57BL/6N* and *B6.129P2(Cg)-Rorc^tm2Litt^/J* [25], *BALB/c DO11.10* TCR-transgenic mice were purchased from either Charles River (Germany) or from The Jackson Laboratory. *C57BL/6-Tcrd-H2BeGFP* mice have been described before [27]. IL-23Rgfp.KI mice have been described previously [30], [46]. Mice were bred and housed under specific pathogen free (SPF) conditions in individually ventilated cages (IVC) either at the Hannover Medical School animal facility or at the SPF facility at the Technical University Munich and all animal experiments were carried out according to institutional guidelines approved by the Niedersächsisches Landesamt für Verbraucherschutz und Lebensmittelsicherheit or by the Bavarian state authorities (permit number 33.9-42502-04-07/1253).

### *In vitro* cytokine stimulation assays

CD4^+^ T cells were isolated from mixed single cell suspensions of spleen and peripheral lymph nodes by MACS cells separation with the CD4^+^ T Cell Isolation Kit (Miltenyi). CD4^+^ cells from *BALB/c DO11.10* TCR transgenic mice were co-cultured for 6–7 days with irradiated (30 Gy) spleen-derived and sex-matched *BALB/c* feeder cells in a ratio of 1×10^6^ DO11.10 to 3×10^6^ *BALB/c* spleen cells per ml. The mouse cytokines TGF-β (2 ng/ml), IL-6 (10 ng/ml), IL-1β (10 ng/ml), IL-21 (10 ng/ml) and TNF-α (10 ng/ml) were purchased from Peprotech. Ovalbumin peptide (Ova_323–339_, 5 µg/ml) was from Anaspec and IL-23 (10 ng/ml) (mouse) from R&D Systems. Human rIL-2 was obtained through the AIDS Research and Reference Reagent Program, Division of AIDS, NIAID, NIH: from Dr. Maurice Gately, Hoffmann – La Roche. For the Th0 condition IL-2 (300 IU/ml) and Ova_322–339_ were added to the co-cultured cells. The Th17 mix contained TGF-β, IL-6, IL-23, IL-1β, TNF-α, anti-IL-4, anti-IFN-γ and Ova_323–339_. The Treg cytokine mix was as follows: IL-2, TGF-β (10 ng/ml), RA (5 nM) and Ova_323–339_. Th1 polarization mix: IL-2, IL-12 (10 ng/ml, Peprotech), anti-IL-4 and Ova_323–339_. Th2 polarization mix: IL-2, IL-4 (20 ng/ml, Peprotech), anti-IFN-γ and Ova_323–339_. Successful polarization was assessed by intracellular staining for the key cytokines IFN-γ, IL-4, IL-17 and the Treg signature transcription factor FoxP3 (data not shown). Anti-IL-4 (clone 11B11, 10 µg/ml) [47] and anti-IFN-γ (clone XMG1.2, 10 µg/ml) [48] were produced in rat hybridoma cell lines, anti-IL-12 (p40/p70, 10 µg/ml) was from BD Pharmingen. Retinoic acid (RA) was obtained from Sigma-Aldrich.

For measurement of intracellular cytokines, T cells were re-stimulated with 50 ng/ml Phorbol-12-myristate-13-acetate (PMA, Calbiochem) and 2 µg/ml ionomycin (Invitrogen) in the presence of brefeldin A (Sigma). Cells were fixed using the BD Cytofix/Cytoperm Kit as described in the supplier's manual.

The IL-17A Secretion Assay (Mouse) was carried out according to the manufacturer's instructions. Briefly, single cell suspensions of spleens and peripheral lymph nodes of *C57BL/6N* mice were stimulated with PMA/ionomycin (50 ng/ml/2 µg/ml) for 3 hours at 37°C with 5% CO_2_. In the next step cells were labelled with the IL-17 Catch Reagent, cultured under rotation at 37°C for 45 min, labelled with the IL-17 Detection Antibody (Biotin) and finally labelled with Anti-Biotin-PE and anti-CD4 (GK1.5) antibody in the presence of FcR block (2.4G2).

### *Ex vivo* FACS cells sorting and FACS analysis

Antibodies directed against αβTCR (clone H57-597), CD4 (clone GK1.5), B220 (clone RA3-GB2), IL-17A (clone ebio17B7), and CCR6 (anti-CD196,) were purchased from ebioscience. Antibodies against CD8α (clone 53-6.7) and CD27 (clone LG.3A10) were obtained from BD Pharmingen. Anti-TCRγδ (clone GL3), CD3 (clone 17A2) and anti-FcR antibody (clone 2.4G2) were produced in rat hybridoma cell lines. FACS analysis was performed using an LSRII flow cytometer (BD Biosciences) and data were collected with BD FACSDiva software (BD Biosciences). FACS sorting was carried out in the central cell sorting facility of the Hannover Medical School on a FACSAriaIIu (BD) or on a MoFlow XDP (Beckman Coulter). Post acquisition analyses and compensation were conducted using FlowJo software 8.8.7 (Treestar). IL-17 producing and non-producing CD4^+^ αβ T cells were sorted from mixed spleen and peripheral lymph node cells from *C57BL/*6*N* mice after revealing IL-17 secreting cells using the mouse IL-17A Secretion Assay (Miltenyi) and subsequent FACS-sorting. γδ T cells [14] were sorted from mixed spleen and peripheral lymph node cells from *C57BL/6-Tcrd-H2BeGFP* mice based on eGFP and CCR6 expression. Rorγt positive and negative cells were sorted from mixed spleen and peripheral lymph node cells from heterozygous *B6.129P2(Cg)-Rorc^tm2Litt^/J* mice based on their GFP expression. IL-23R expressing or non-expressing cells were sorted according to their GFP expression from either homozygous ( = IL-23R^−/−^) or heterozygous IL-23Rgfp.KI mice. Human samples were sorted as follows: To capture cytokine expressing cells, PBMC were enriched for CD4^+^ T cells using negative selection magnetic beads (Stemcell technologies) and stimulated for 2 hours with PMA (10 ng/ml, Sigma) and ionomycin (1 µg/ml, Sigma). IL-17A and IFN-γ secreting cells were detected by a human Secretion Assay, Miltenyi Biotec, according to manufacturer's instructions and co-stained for CD4 (CD4-PC7, Beckman Coulter).

### Quantitative Real-Time PCR (qRT-PCR)

*In vitro* cultured or FACS sorted cells were resuspended in 700 µl QIAzol lysis buffer, if not described differently and total RNA with conservation of small RNAs was isolated by QIAgen miRNeasy Kit. Quantitative Real-Time PCR was carried out using probes for hsa-miR-133b or hsa-miR-206 (Applied Biosystems, Taqman miRNA Assays ID 00510 and ID 002247) on an Applied Biosystems StepOnePlus Real-Time PCR System. The validity of the qRT-PCR assays for miR-133b and miR-206 was assessed by overexpression and subsequent detection of these in BWα^−^β^−^ cells (Fig. S5). Fold-difference was calculated by the ΔΔC_t_ method normalized to snoRNA412 as housekeeping gene for mouse samples or RNU48 for human samples (both Applied Biosystems Assay ID 001243 and ID 001006). The SD values for the representative experiments were calculated from either triplicates or quadruplicates of the combined coefficients of variation from sample and calibrator according to the manufacturer's manual (Applied Biosystems).

### ELISA

Secreted IL-17A protein was detected by standard sandwich ELISA purchased from BioLegend. Concentrations of IL-17A were calculated using recombinant purified IL-17A protein. Standard curve and sample concentrations were calculated based on the mean of triplicates for each dilution or sample.

### Retroviral infections

The retroviral constructs encoding mmu-miR-133b and mmu-miR-206 were generated by inserting the respective pre-miRNA sequences flanked by approximately 125 bp into the 3′LTR of the vector MDH-PGK1-GFP_2.0 (Addgene, [35]). miRNA sequences were obtained by PCR from the BAC AC159614 with the following primers:

206-forward: CCGGTTAACTCGAGGATAGTTTCTGGGAGTGTAG;
206-reverse: CTAGCTAGGAATTCGGAGAAGTCTGTAGAGGTAG;
133b-forward: CCGGTTAACTCGAGCTCTGTGAGAGGTTAGTCAG;
133b-reverse: CTAGCTAGGAATTCTGTGACCTGTGAACTTGGGC.

The resulting vectors encode GFP under control of the PGK promoter and miRNA under control of the human H1 promoter. Retroviral supernatants were generated by transient transfections of 293T cells with these retroviral constructs and the pCLEco packaging plasmid (Imgenex). Lineage negative BM cells were retrovirally transduced as described [49] and intravenously injected into irradiated (9 Gy) syngeneic hosts. The resultant chimeric mice were analyzed after 8–10 weeks. BW5147α-β- thymoma cells or CD4^+^ T cells from DO11.10-TCR transgenic mice were transduced using standard spin infection and successfully transduced cells were identified based on GFP expression.

### Statistics

Statistical analysis was performed using GraphPad Prism software using the two-tailed unpaired Student's t-test. P-values<0.05 were considered as significant (*), <0.01 (**), <0.001 (***). If not otherwise described error bars are calculated from SEM of n = 3 experiments.

## Supporting Information

Figure S1 **Mitogenic stimulation of** ***in vitro*** **Th17 polarized cells does not change the expression level of miR-133b or miR-206. Related to** **Figure 1D and 1E** **.** The same protocol as in Figure 1 was used for the polarization of T cells to the Th17 lineage. 50% of the cells were additionally stimulated with PMA/ionomycin for the last 3 h before harvesting. Expression levels for miR-133b and miR-206 were compared by qRT-PCR relative to Th0 (control). One representative experiment is shown out of two independent experiments with similar results with 2 mice per experiment. Error bars represent SD values.
(TIFF)

Figure S2 **The activation status of CD4^+^ T cells and γδ T cells does not influence miR-133b and miR-206 expression. Related to** **Figure 2** **and** **Figure 3** **.** (A) CD4^+^ cells were sorted into naive CD4^+^ T cells (CD44^lo^, CD62L^hi^) and activated/memory CD4^+^ T cells (CD44^hi^ CD62L^lo^) from lymph node and spleen cells of *C57BL/6N* mice and compared for their expression levels of miR-133b and miR-206 as described. Shown is one experiment with 5 mice. (B) and (C) γδ T cells were sorted into CCR6^+^, CCR6^−^ CD44^hi^ and CCR6^−^ CD44^lo^ cells from lymph node and spleen cells of *TcrdH2BeGFP* mice and compared for their expression levels of miR-133b and miR-206. Shown is one representative experiment of two independent experiments with similar results with 4–6 mice per experiment. Error bars show SD values of 3 replicates.
(TIFF)

Figure S3 **Expression of** ***Il17a*** **mRNA in skeletal muscle compared to peripheral lymph node cells and Th17 polarized cells.** Skeletal muscle (tibia), peripheral lymph node (inguinal) and Th17 polarized cells were compared for their *Il17a* mRNA expression by Taqman Real-Time PCR. Shown are the results of 2 independent experiments from 1 mouse per group.
(TIFF)

Figure S4 **No regulation of the predicted target gene Ets1 by miR-133b or miR-206.** Luciferase assay using the psiCHECK-2 vector (Promega) into which the Ets1 3′-UTR was cloned downstream of Renilla luciferase. By nucleofection with the Amaxa-nucleofection reagent the psiCHECK-2-Ets1 vector was introduced into the BW5147 α-β- cell line that was stably transduced with either the empty MDH1-PGK-GFP2.0 (Addgene) plasmid, the latter plasmid with miR-133b or with miR-206.
(TIFF)

Figure S5 **Proof of principle for the miRNA Real-Time detection system.** The miRNAs mmu-miR-133b and mmu-miR-206 were cloned from the BAC AC159614 with the primers mentioned in Materials and Methods. Both miRNAs were introduced into the retroviral vector MDH1-PGK-GFP_2.0 (Addgene) with restriction enzymes *EcoRI* and *XhoI*. The respective constructs were then transfected into 3T3 cells via Calcium-Phosphate Transfection Kit (Sigma) and the supernatant was used for the stable transfection of the BW5147α-β- thymoma cell line.
(TIFF)
