# Supplementary figures and images for: Expression of miRNAs miR-133b and miR-206 in the Il17a/f Locus Is Co-Regulated with IL-17 Production in αβ and γδ T Cells
Source: PLoS One. 2011 May 26;6(5):e20171. doi: 10.1371/journal.pone.0020171 (PMC3102691; doi:10.1371/journal.pone.0020171)

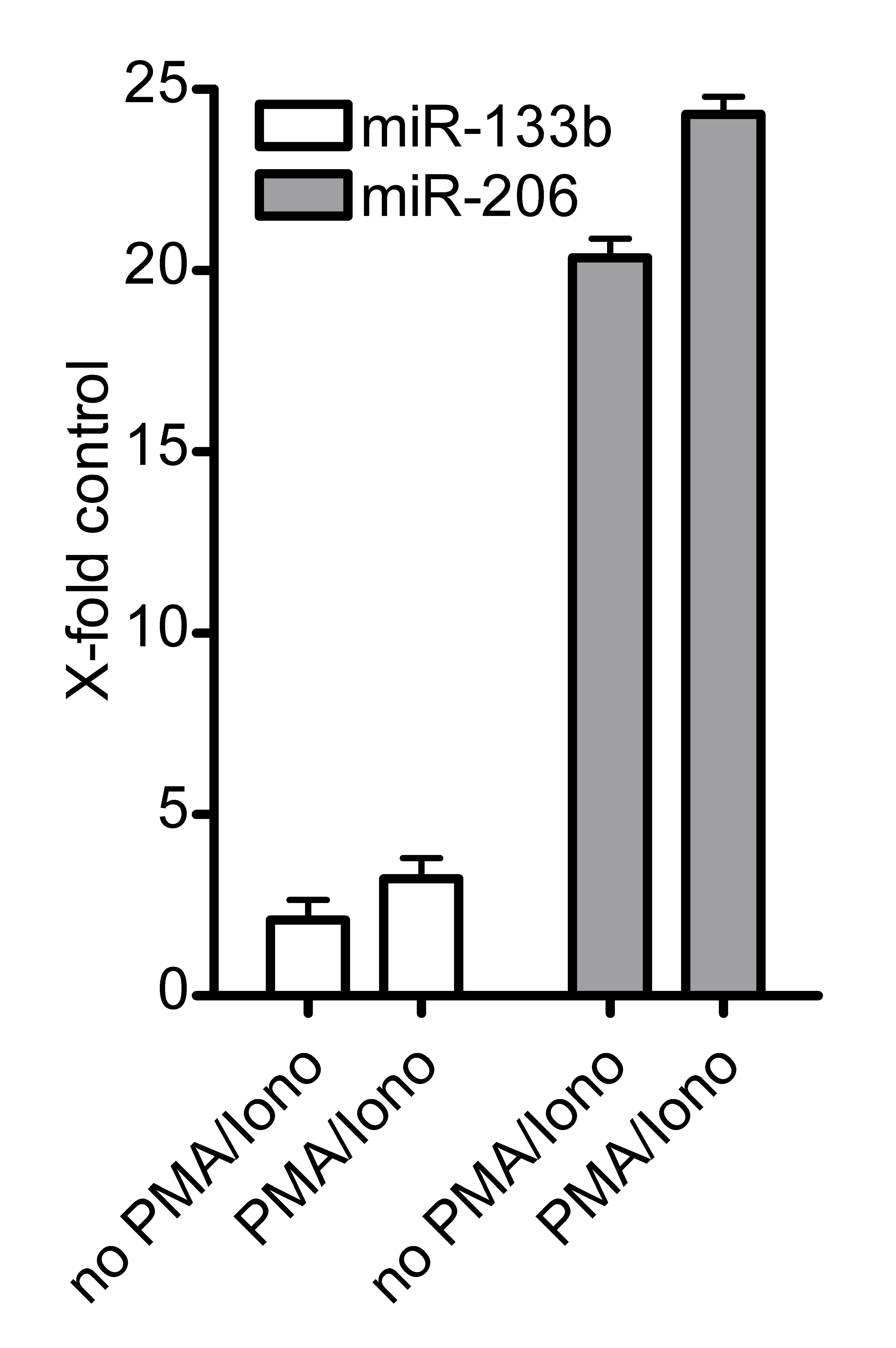

Supplement: Figure S1 — Mitogenic stimulation of in vitro Th17 polarized cells does not change the expression level of miR-133b or miR-206. Related to Figure 1D and 1E . The same protocol as in Figure 1 was used for the polarization of T cells to the Th17 lineage. 50% of the cells were additionally stimulated with PMA/ionomycin for the last 3 h before harvesting. Expression levels for miR-133b and miR-206 were compared by qRT-PCR relative to Th0 (control). One representative experiment is shown out of two independent experiments with similar results with 2 mice per experiment. Error bars represent SD values. (TIFF) [file pone.0020171.s001.tif]

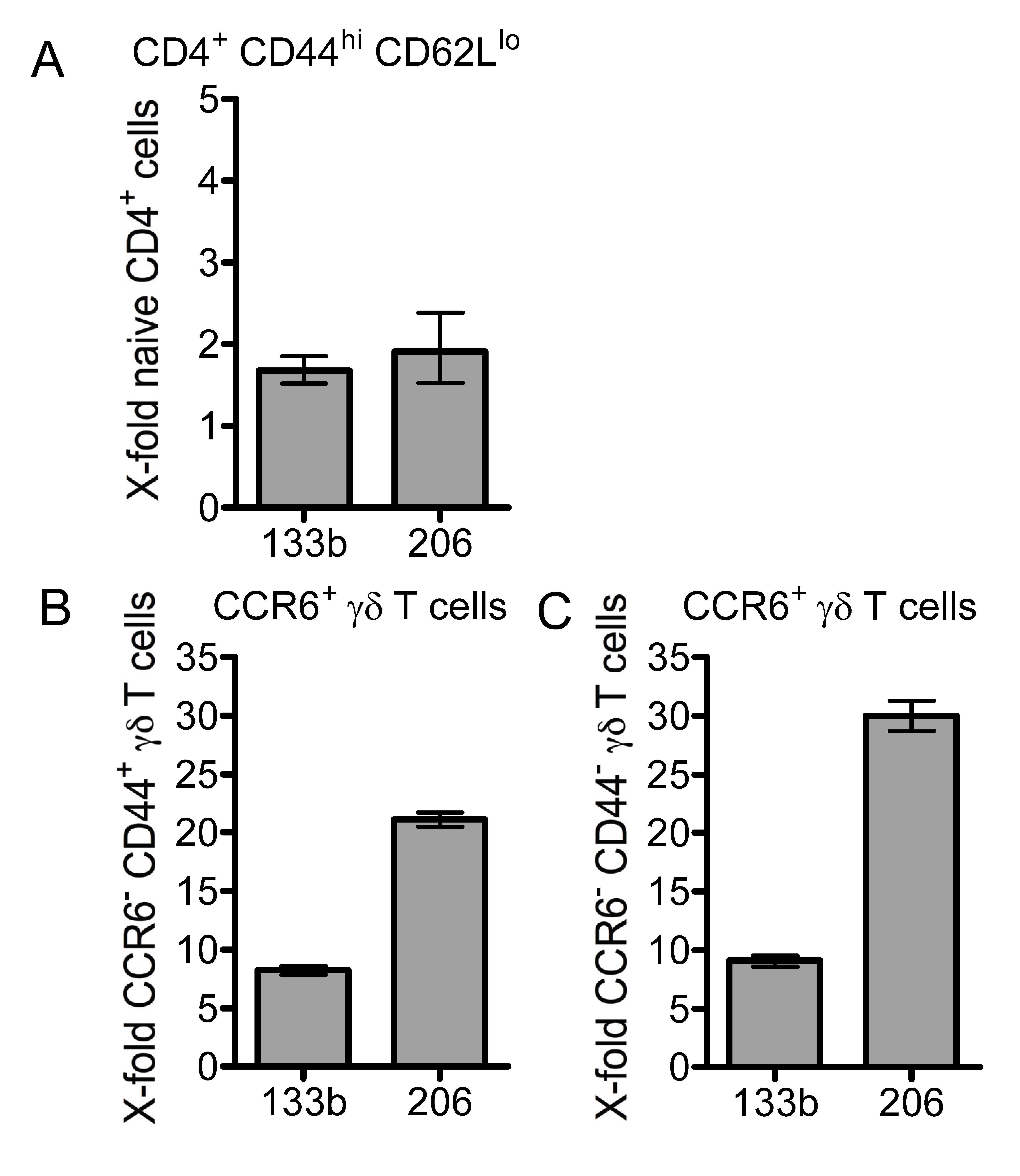

Supplement: Figure S2 — The activation status of CD4+ T cells and γδ T cells does not influence miR-133b and miR-206 expression. Related to Figure 2 and Figure 3 . (A) CD4+ cells were sorted into naive CD4+ T cells (CD44lo, CD62Lhi) and activated/memory CD4+ T cells (CD44hi CD62Llo) from lymph node and spleen cells of C57BL/6N mice and compared for their expression levels of miR-133b and miR-206 as described. Shown is one experiment with 5 mice. (B) and (C) γδ T cells were sorted into CCR6+, CCR6− CD44hi and CCR6− CD44lo cells from lymph node and spleen cells of TcrdH2BeGFP mice and compared for their expression levels of miR-133b and miR-206. Shown is one representative experiment of two independent experiments with similar results with 4–6 mice per experiment. Error bars show SD values of 3 replicates. (TIFF) [file pone.0020171.s002.tif]

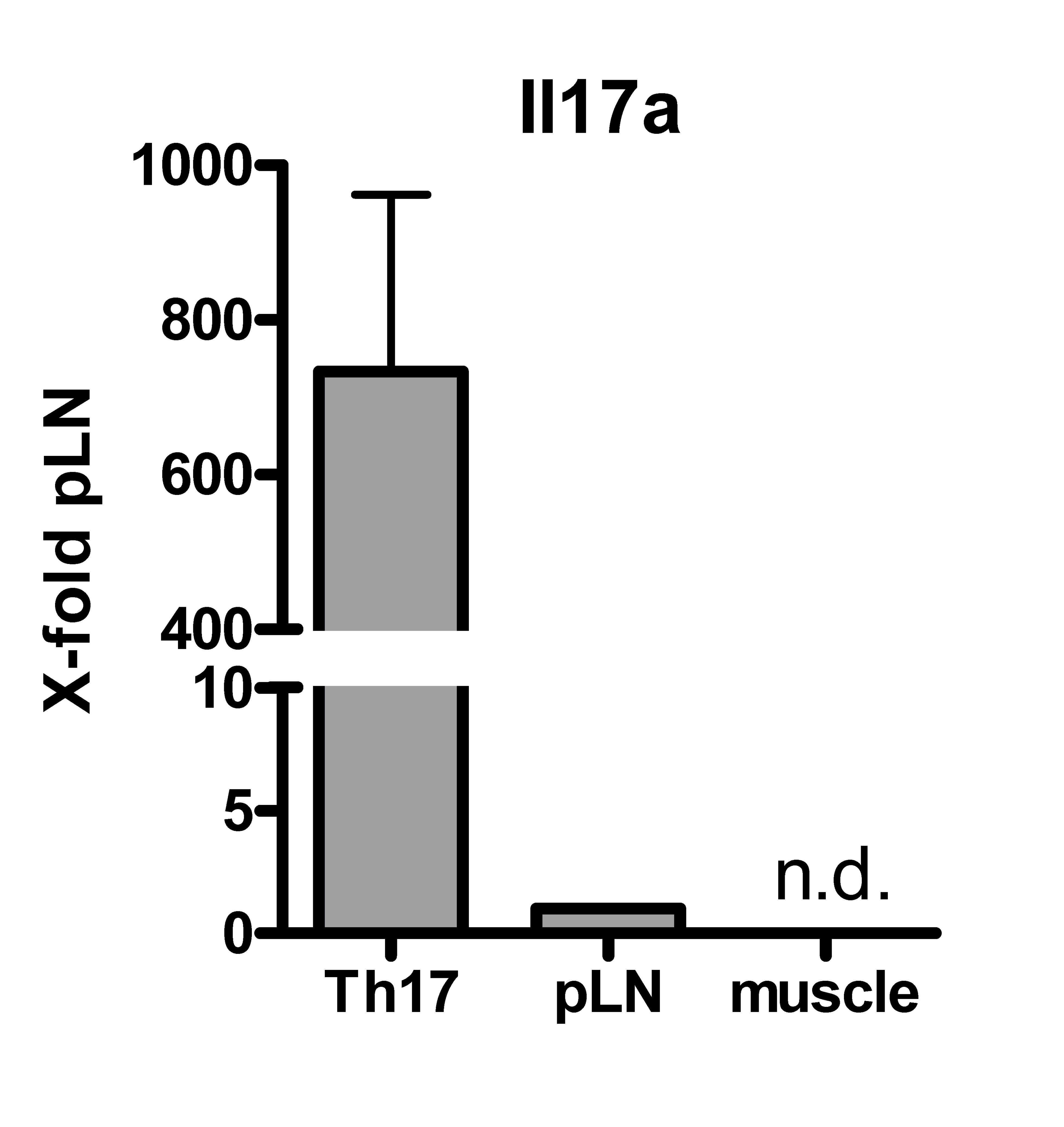

Supplement: Figure S3 — Expression of Il17a mRNA in skeletal muscle compared to peripheral lymph node cells and Th17 polarized cells. Skeletal muscle (tibia), peripheral lymph node (inguinal) and Th17 polarized cells were compared for their Il17a mRNA expression by Taqman Real-Time PCR. Shown are the results of 2 independent experiments from 1 mouse per group. (TIFF) [file pone.0020171.s003.tif]

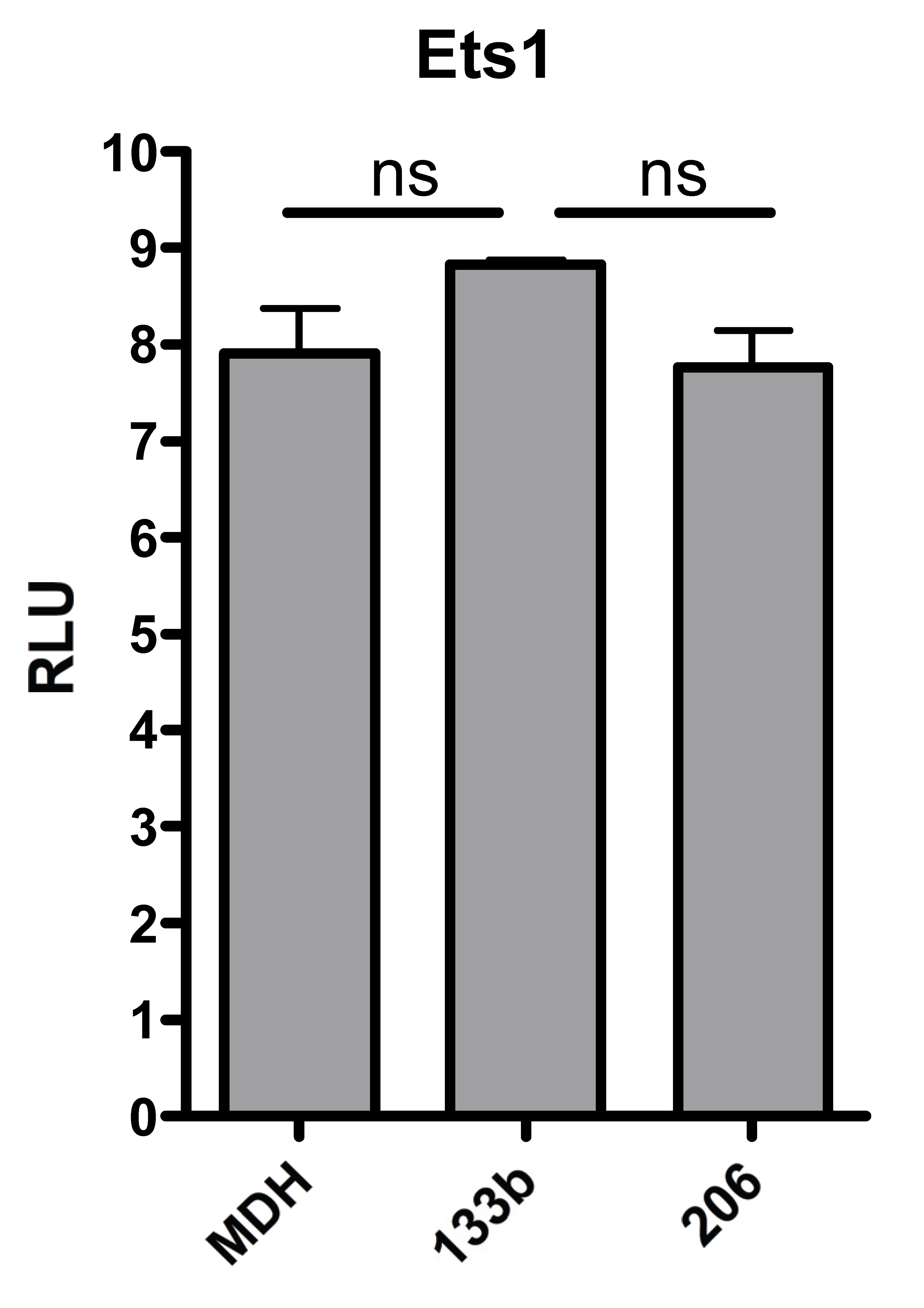

Supplement: Figure S4 — No regulation of the predicted target gene Ets1 by miR-133b or miR-206. Luciferase assay using the psiCHECK-2 vector (Promega) into which the Ets1 3′-UTR was cloned downstream of Renilla luciferase. By nucleofection with the Amaxa-nucleofection reagent the psiCHECK-2-Ets1 vector was introduced into the BW5147 α-β- cell line that was stably transduced with either the empty MDH1-PGK-GFP2.0 (Addgene) plasmid, the latter plasmid with miR-133b or with miR-206. (TIFF) [file pone.0020171.s004.tif]

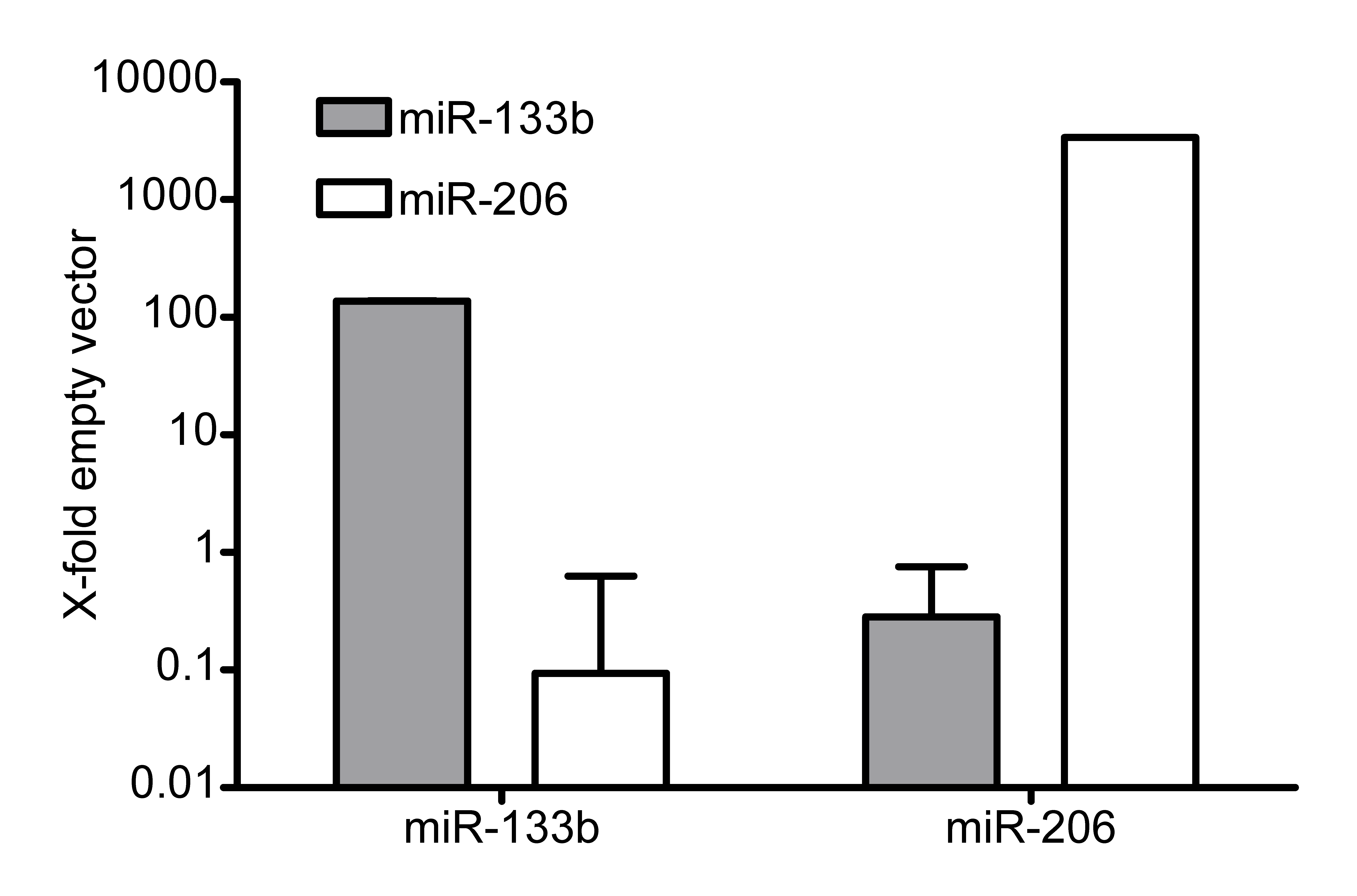

Supplement: Figure S5 — Proof of principle for the miRNA Real-Time detection system. The miRNAs mmu-miR-133b and mmu-miR-206 were cloned from the BAC AC159614 with the primers mentioned in Materials and Methods. Both miRNAs were introduced into the retroviral vector MDH1-PGK-GFP_2.0 (Addgene) with restriction enzymes EcoRI and XhoI. The respective constructs were then transfected into 3T3 cells via Calcium-Phosphate Transfection Kit (Sigma) and the supernatant was used for the stable transfection of the BW5147α-β- thymoma cell line. (TIFF) [file pone.0020171.s005.tif]
